# A rapid, inexpensive, and semi-quantitative method for determining pollen tube extension using fluorescence

**DOI:** 10.1186/1746-4811-10-3

**Published:** 2014-01-23

**Authors:** Emily Hartman, Clara Levy, David M Kern, Mark A Johnson, Amit Basu

**Affiliations:** 1 Department of Chemistry, Brown University, Providence, RI 02912, USA; 2 Department of Molecular Biology, Cell Biology, and Biochemistry, Brown University, Providence, RI 02912, USA

**Keywords:** Pollen tube growth, Chemical genetics, Chemical genomics, Chemical screening

## Abstract

**Background:**

Pollen tubes extend rapidly when pollen grains are incubated in defined media. Tube extension requires many critical functions of plant cells including molecular signaling, cytoskeleton remodeling, secretion, and cell wall synthesis. Consequently, pollen tube growth has been established as a way to conduct primary screens of chemical libraries to identify compounds that perturb key cellular processes in plants.

**Results:**

Here we report a simple, inexpensive, rapid and semi-quantitative method for measurement of pollen tube growth in microtiter plates. The method relies on Congo Red binding to pollen tubes and correlates dye fluorescence to tube length.

**Conclusions:**

This method can be used in any laboratory without specialized equipment, and has the potential to enable larger screens as chemical libraries grow and to make chemical screening accessible to researchers building specialized libraries designed to probe pathways in plant biology.

## Background

Small molecules that modulate biological activity have been used in chemical genetic screens to reveal molecular mechanisms underpinning many biological processes in a broad array of organisms [1-3]. Chemical genetics complements molecular genetics as a discovery tool because small molecules can overcome genetic redundancy and lethality, and can provide enhanced temporal and dosage control over the phenotype of interest. The application of chemical genetic strategies in plant biology have resulted in numerous notable successes, including the discovery of several molecules that target specific auxin or brassinosteroid signaling pathways, modulators of cellulose synthase dynamics, and the identification of the long sought-after receptor for abscisic acid, a plant hormone that regulates stress responses [4-7]. Broad application of chemical genetics requires the development of rapid and inexpensive chemical screening methods that can be applied to multiple species. Ideally, primary-screening methods would be quantitative and would interrogate a broad spectrum of cellular activities so that compounds could be identified for further study in more directed screens, especially as newer and larger compound collections designed for plant chemical genetics are generated.

The pollen tube is an attractive system for chemical genetics in plants because it is a single cell that extends rapidly in a simple defined media. Unlike other plant cells, pollen grains are easily collected from the plant without invasive procedures, and pollen from many species can be stored for future use. Moreover, pollen tubes extend rapidly, reaching lengths in 4 hours that are 10 times the initial grain diameter. This rapid extension occurs by tip growth, a process that relies on many basic cellular functions including actin-myosin-mediated vesicle trafficking [8], calcium gradients and flux [9], GTPase activity [10], kinase signaling [11], and cell wall biosynthesis [12]. Pollen tube length is most commonly determined using microscopy, which requires time-consuming scoring and measurement of individual pollen tubes. Alternative approaches for indirectly quantifying tube length, such as turbidity measurements or ELISAs, have been reported, but each of these require additional processing that reduces their attractiveness for chemical genetic screens [13,14].

The potential of the pollen tube for chemical screening was demonstrated by pioneering work from the Raikhel laboratory [15,16]. They used automated confocal microscopy in a mictotiter format to determine whether small molecules inhibited the germination of tobacco pollen. This primary chemical screen was applied to multiple chemical libraries containing more than 48,000 compounds, identifying almost 500 active lead compounds. In a secondary screen they analyzed images to determine whether the lead compounds altered the normal localization of a GFP-labeled protein to the tip of a growing pollen tube with a functioning secretory system. One secondary screen identified a compound that inhibits endocytosis in Arabidopsis seedlings [15]. These experiments illustrate that primary screening of tobacco pollen tube growth is a powerful approach to identify biologically active molecules that can then be analyzed by subsequent assays in other species and more focused cellular contexts. However, the screening method is resource intensive, requiring a highly specialized automated confocal imaging system. Our aim was to develop a method to measure pollen tube extension that would achieve two goals: 1) provide a semi-quantitative readout of relative pollen tube extension that is amenable to screening of large chemical libraries; and 2) make chemical screening more accessible so that biologists can routinely carry out library screening in their own laboratories, both with commercially available libraries as well as in-house compound collections.

We report here the use of Congo Red, a polysaccharide binding dye [17,18] which binds to pollen [19], to develop a semi-quantitative pollen tube extension assay that works in microtiter plate format and can be analyzed using a simple fluorescence plate reader. Increases in Congo Red fluorescence correlate with increases in pollen tube length. When compounds that limit pollen tube extension are present in the wells, lower levels of fluorescence are observed. The assay is inexpensive, rapid, and is sensitive across a range of pollen tube lengths.

## Results and discussion

**Figure 1:**
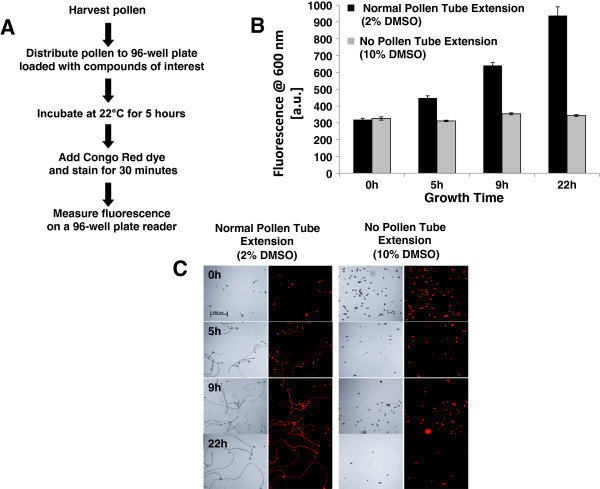
**Congo Red fluorescence correlates with pollen tube extension. A)** Schematic of workflow for measuring pollen tube extension using Congo Red. **B)** Fluorescence (arbitrary units; excitation: 470 nm, emission: 600 nm) is plotted against incubation time for pollen incubated in 2% DMSO (normal pollen tube extension) or 10% DMSO (no pollen tube extension, grains only). Error bars represent the standard deviation of at least three replicate experiments. **C)** Micrographs (DIC, left; Fluorescence, right) of pollen incubated in 2% or 10% DMSO for the indicated times (h, hours) and stained with Congo Red.

A workflow diagram outlining the execution of the assay is shown in Figure 1A. We harvested pollen from flowering tobacco plants, then incubated the pollen grains in a 96-well microtiter plate in the presence of pollen growth medium (PGM). After a defined period of pollen tube extension (see Figure 1B), we added Congo Red and incubated the pollen tubes with the dye for 30 minutes. A fluorescence plate reader was used to measure the absolute emission in each well.

Pollen grains were incubated in PGM for 0, 5, 9, and 22 hours. As most chemical libraries are formulated as stock solutions in DMSO, we used 2% DMSO as our vehicle control. Under these conditions Congo Red fluorescence increased as the tubes extended over time (Figure 1B, black bars). In contrast, the inclusion of 10% DMSO completely inhibited pollen germination and Congo Red fluorescence did not change with incubation time (Figure 1B, gray bars). Microscopy showed that Congo Red fluorescence correlated with pollen tube extension (Figure 1C). These observations indicated that Congo Red fluorescence could be used as a proxy for pollen tube extension. Subsequent fluorescence experiments were carried out after 5 hours growth, as this time point provided a sufficient dynamic range to determine relative pollen tube extension (Figure 1B).

**Figure 2:**
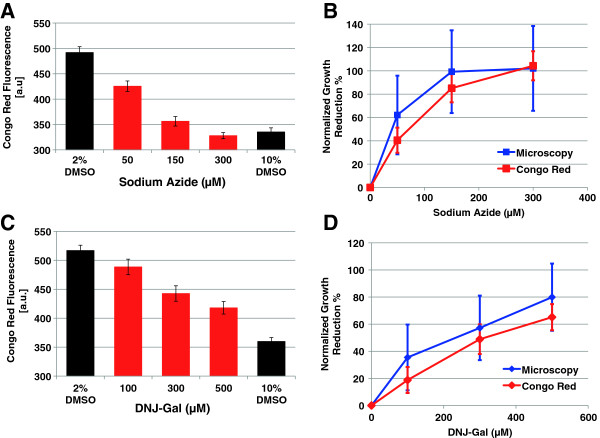
**Congo Red fluorescence provides a semi-quantitative proxy for pollen tube extension.** The effect of sodium azide **(A-B)** or DNJ-Gal on pollen tube extension (5 hours) was measured using Congo Red fluorescence **(A,C)** or microscopy **(B,D)**. **A,C)** Fluorescence (arbitrary units; excitation: 470 nm, emission: 600 nm) is plotted against concentration of compound, 2% DMSO and 10% DMSO controls are included. Error bars represent the standard deviation of at least three replicate experiments. **B,D)** To compare Congo Red fluorescence with microscopy measurements, the % reduction in tube extension compared to the 2% DMSO control was calculated for each method and was plotted against compound concentration. Error bars for tube length are based on the standard deviations of the mean tube lengths measured in at least two separate experiments.

Ideal primary screens for chemical activity would be quantitative so that the relative activity of a series of compounds could be determined. We used varying concentrations of the metabolic inhibitor sodium azide to determine whether Congo Red fluorescence could yield information about relative pollen tube extension. Decreasing Congo Red fluorescence is observed in the presence of increasing sodium azide concentrations (Figure 2A). At 300 μM sodium azide, the fluorescence intensity is comparable to that of the no germination control (10% DMSO). We determined the average pollen tube length in each well using microscopy in order to determine the accuracy of the Congo Red fluorescence values. To facilitate the comparison of fluorescence [a.u.] and microscopy [μM] data, we converted both tube length values and fluorescence values to % Growth Reduction, a measurement that standardizes normal extension to 0% Growth Reduction and no germination to 100% Growth Reduction. This allowed us to better compare the relative inhibition from both data sets. At each concentration of azide examined, a strong correlation was observed between the Growth Reduction percentages derived from tube length and fluorescence measurements (Figure 2B).

Pollen tube extension involves rapid assembly and remodeling of the cell wall [12]. Tube extension is accompanied by high levels of polysaccharide processing enzymes such as pectinases and other glycosyl hydrolases. β-galactosidases are amongst the most abundant glycosyl hydrolases expressed in Arabidopsis pollen tubes and β-galactosidase activity has been detected during pollen grain development in tobacco [20,21]. Deoxygalactonijirimycin (DNJ-Gal) is a readily available alkaloid that inhibits both α and β-galactosidases [22]. We determined the effect of this inhibitor (% reduction of normalized growth, see methods) on pollen tube extension using Congo Red fluorescence and by microscopy and found an inverse relationship between tube length and the DNJ-Gal concentration. Again, Congo Red fluorescence and microscopy yielded similar results, and allowed us to differentiate between the activity of different concentrations of this glycosidase inhibitor (Figure 2B, D).

Inhibition of pollen tube length determined via direct microscopy was consistently greater than that determined by measuring total Congo Red fluorescence (Figure 2D). This is likely to be the case for compounds that affect pollen germination or those that affect both pollen germination and pollen tube extension because pollen grains that do not germinate do not contribute to changes in Congo Red fluorescence intensity. This leads to a decrease in measured growth reduction by Congo Red fluorescence relative to direct measurement of pollen tube lengths, a measurement that ignores ungerminated pollen grains. Indeed, inspection of germination percentages for the data shown in Figure 2D indicates a decrease from 70% germination for the full growth control to approximately 60% in each of the DNJ-Gal containing wells. Thus, it is important to carry out follow up experiments on compounds of interest to determine whether changes in Congo Red fluorescence are due primarily to inhibition of pollen germination or pollen tube extension. For the same reason, it is also important to note that this method may not be appropriate to compare pollen tube growth rates among different populations of pollen with different basal pollen germination rates. For example, the method would not be useful for identifying compounds that differentially affect pollen tube extension in mutants with germination defects compared to wild type pollen.

We developed a pollen tube growth assay that can be carried out in microtiter plates and provides quantitative chemical activity data via a fluorescence plate reader (Figure 1). The dynamic range of the assay is sufficient to differentiate between strong and weak inhibition after five hours of pollen tube growth (Figures 1B and 2), which allows assays to be completed without overnight incubation. However, we note that additional growth time increases the dynamic range without a significant increase in error (Figure 1B), which may be useful for identifying weak inhibitors or compounds that require additional time to penetrate cells. Even though we find that increases in Congo Red fluorescence between 9 and 22 hours of incubation are accompanied by increases in pollen tube extension (Figure 1C), caution should be used when extending the assay beyond 9 hours because prolonged growth of pollen tube in vitro is associated with loss of tip polarity, pollen tube rupture, and other growth defects.

The main strengths of this assay are that it is rapid, inexpensive, simple, and quantitative. However it has two obvious drawbacks. First, fluorescent compounds have the potential to interfere with the assay and inhibitory molecules that emit at 600 nm may present as false negatives. This can be addressed by pre-screening plates of molecules before running pollen tube growth assays. The other drawback is that the assay does not provide information on pollen tube morphology. For example, molecules that disrupt the secretory pathway [15] or the signaling pathways that define the pollen tube tip [16] are expected to produce tubes that bulge rather than extend with a uniform diameter as seen in Figure 1C. This limitation can be addressed in part by designing secondary screens using microscopy, although some compounds may be missed that affect morphology without affecting Congo Red fluorescence.

## Conclusions

This assay, developed here using tobacco pollen, should be transferable to any species with pollen that can be readily collected and grown in vitro. For example, robust protocols have been developed for tomato [23] and maize [24] pollen tube growth, which opens the possibility of conducting comparative chemical screens. Arabidopsis pollen will be more challenging because large numbers of plants are required to produce the amount of pollen necessary for large-scale screening projects [25]. Finally, we have shown that DNJ-Gal, a broad-spectrum inhibitor of galactosidase activity, has an inhibitory effect on pollen tube elongation. This suggests a role for these enzymes in pollen tube extension and warrants further screening of libraries containing molecules with similar or related structures to find more potent inhibitors that can be used to identify critical molecular targets.

## Methods

### Collection and delivery of tobacco pollen into microtiter plates

Dehiscent anthers from ten freshly opened *Nicotiana tabacum* cultivar Petit Havana SR1 flowers were collected in 15 ml conical tubes by cutting the corolla with dissection scissors approximately 1 cm up from the receptacle and allowing anthers to drop into the tube. The tube was filled with 5 mL 1X pollen growth medium (PGM: 9% w/v sucrose, 0.005% w/v boric acid, 0.5 mM CaCl_2_, 0.5 mM Ca(NO_3_)_2_, 0.5 mM MgSO_4_), capped, and inverted 5-6 times to suspended pollen grains in PGM. The PGM containing pollen and anthers was poured through an 80 μm nytex filter and the filtrate was collected in a multichannel pipette trough. The anthers and vial were washed with another 5 mL of PGM. The trough was tipped back and forth several times to suspend the pollen evenly in the PGM. A multichannel pipette was used to add 50 μL of the suspension to each row of the microtiter well plate (see below), taking care to repeatedly agitate the trough before each delivery to ensure even pollen distribution from row to row.

### Preparation of 96 well flat-bottomed microtiter plates for small molecule screening

Each row of the plate contained at least one well with a pollen tube extension positive control (2% DMSO) and a no germination negative control (10% DMSO) so that row-to-row variability can be internally normalized. Compounds of interest were dissolved in DMSO or water and 2 μL of the compound stock solution was added to the well. In all cases the total amount of DMSO in the well was kept at 2 μL, except for the no germination control, which contained 10 μL DMSO. The well was filled to a total volume of 25 μL by the addition of 23 μL water (15 μL in the case of the no germination control). This was followed by the addition of 50 μL of the tobacco pollen as described above. Finally, an additional 25 μl of 2X PGM (18% w/v sucrose, .01% w/v boric acid, 1 mM CaCl_2_, 1 mM Ca(NO_3_)_2_, 1 mM MgSO_4_) was added for a total well volume of 100 μl, consisting of 1X PGM, 2% DMSO, and pollen. The normalized growth reduction % was calculated using the following formula:

$$\begin{array}{l} \mathrm{Normalized}\;\mathrm{Growth}\;\mathrm{Reduction}\\ \;=\left[F_{2\%\;\mathrm{DMSO}}–F_{\mathrm{sample}}\right]/\left[F_{2\%\;\mathrm{DMSO}}–F_{10\%\;\mathrm{DMSO}}\right]\times 100\% \end{array}$$

### Incubation

The microtiter well plates were covered with a lid and incubated in a Tupperware or glass container with wet paper towels and water to maintain 100% humidity. Incubation was carried out at 22°C for 5 hours. After incubation, 100 μL of a 711 μM aqueous solution of Congo Red (disodium salt) was added to each well. After 30 minutes of incubation with the dye, the plate was read on a Tecan Safire plate reader (λ excitation-470 nm; λ emission-600 nm).

### Microscopy

To determine whether fluorescence intensity correlated with pollen tube length, 20 μL of the contents of each microtiter wells were removed using a wide-bore pipette. The solution was added directly to a microscope slide within borders marked by a hydrophobic barrier pen. Pollen growth media was removed using a pipette, and 10 μL of 60% (v/v) glycerol/water solution was added to the slides in order to arrest pollen tube growth. Images of the pollen tubes were taken using differential interference contrast (DIC) optics on a Zeiss Axiovert 200 M fluorescent microscope (Carl Zeiss, Oberkochen, Germany). Pollen tube germination and length was quantified using ImageJ software. Two replicates of each well of pollen were placed on each slide. 50 pollen tubes and 100-300 grains were measured for tube length and germination respectively per experiment. Experiments were repeated three times.

## Abbreviations

DIC: Differential interference contrast; DMSO: Dimethylsulfoxide; DNJ-Gal: Deoxygalactonijirimycin; PGM: Pollen growth medium.

## Authors’ contributions

EH, DK, MJ, and AB designed the method and experiments to test it. EH and DK carried out Congo Red binding experiments and fluorescence measurements. EH and CL carried out pollen tube measurements via microscopy. EH, MJ, and AB drafted and edited the manuscript. All authors read and approved the final manuscript.
